# Production of a New Biosurfactant by a New Yeast Species Isolated from *Prunus mume* Sieb. *et* Zucc.

**DOI:** 10.4014/jmb.2205.05052

**Published:** 2023-05-04

**Authors:** Jeong-Seon Kim, Miran Lee, Dae-Won Ki, Soon-Wo Kwon, Young-Joon Ko, Jong-Shik Kim, Bong-Sik Yun, Soo-Jin Kim

**Affiliations:** 1 Agricultural Microbiology Division. National Institute of Agricultural Science, Rural Development Administration, Wanju-gun, Jeollabuk-do 55365, Republic of Korea; 2 Division of Biotechnology and Advanced institute of Environmental and Bioscience, College of Environmental and Bioresource Sciences, Jeonbuk National University, Iksan-si, Jeollabuk-do 54596, Republic of Korea; 3 Marine industry research institute for east sea rim, Uljin-gun, Geongsangbuk-do 36315, Republic of Korea

**Keywords:** Yeast, novel species, new biosurfactant, chemical structure

## Abstract

Biosurfactants reduce surface and interfacial tension due to their amphiphilic properties and are an eco-friendly alternative for chemical surfactants. In this study, a new yeast strain JAF-11 that produces a biosurfactant was selected using drop collapse method, and the properties of the extracts were investigated. The nucleotide sequences of the strain were compared with closely related strains and identified based on the D1/D2 domain of the large subunit ribosomal DNA (LSU) and internal transcribed spacer (ITS) regions. *Neodothiora populina* CPC 39399^T^, the closest species with strain JAF-11, showed a sequence similarity of 97.75% for LSU and 94.27% for ITS, respectively. The result suggests that the strain JAF-11 represents a distinct species that cannot be assigned to any existing genus or species in the family *Dothideaceae*. Strain JAF-11 produced a biosurfactant reducing the surface tension of water from 72 mN/m to 34.5 mN/m on the sixth day of culture and the result of measuring the critical micelle concentration (CMC) by extracting the crude biosurfactant was found to be 24 mg/l. The molecular weight 502 of the purified biosurfactant was confirmed by measuring the fast atom bombardment mass spectrum. The chemical structure was analyzed by measuring ^1^H nuclear magnetic resonance (NMR), ^13^C NMR, and two-dimensional NMRs of the compound. The molecular formula was C_26_H_46_O_9_, and it was composed of one octanoyl group and two hexanoyl groups to *myo*-inositol moiety. The new biosurfactant is the first report of a compound produced by a new yeast strain, JAF-11.

## Introduction

The surfactants have amphiphilic properties possessing both non-polar (hydrophobic) and polar (hydrophilic) moieties that allow reducing the surface and interfacial tension between biphasic systems as a liquid-liquid interface or solid-liquid boundaries [1, 2]. Therefore, surfactants are one of the important compounds having the potential commercial application in detergents, cosmetics, food ingredients, agricultural, pharmaceutical, paint, textile and paper etc. [3, 4]. However, surfactants are mainly chemically synthesized from petroleum-based resources, which can cause environmental problems due to toxicity [5]. As environmental awareness has gradually increased over the past decades, the demand for eco-friendly compounds has increased, and accordingly, there is an increasing interest in microbial biosurfactants [6]. In particular, microbe-derived biosurfactants have advantages such as environmental compatibility, activity, stability, and lower toxicity than chemically synthesized equivalents [7-11].

Microbial biosurfactants are classified according to ionic charges, molecular weight, secretion type, and chemical structure, and various biosurfactants are produced depending on the type of microorganisms [12]. Among them, glycolipids have been best known as a class of biosurfactants with the highest commercial importance [13], and the microbe-derived glycolipids biosurfactant rhamnolipids were described for the first time in 1946 [14]. Despite their high productivity, most of them are produced by the opportunistic pathogen *Pseudomonas aeruginosa* [15]. The most promising biosurfactant sophorolipids are produced by non-pathogen yeast *Starmerella bombicola*, *Candida stellate*, *C. riodocensis*, *C. apicola*, *C. batistae*, *C. kuoi*, and *C. floricola* [16, 17]. Furthermore, mannosylerythritol lipids (MEL) and trehalolipids have been isolated from *Pseudozyma* and *Rhodococcus erythopolis* [18].

In the 20^th^ century, research on biosurfactants focused on understanding and optimizing the production process. Since then, research on utilizing various renewable resources, discovering new producer strains, or developing genetically modified strains has been reported [6]. Since biosurfactants have a various functional properties depending on their chemical structure [19], it is necessary to continuously secure a variety of biosurfactants to be applied to various fields. The present study is the first report of a new biosurfactant extracted from the new yeast strain JAF-11. The chemical structure of the biosurfactant extracted from the yeast has been characterized and identified as a new biosurfactant by nuclear magnetic resonance spectrometry techniques.

## Materials and Methods

### Isolation and Screening of Biosurfactant Producing Yeast

Several strains of yeast from flowers in various regions of the Republic of Korea were isolated and purified through the cultivation at 25°C on YPD medium (yeast extract peptone dextrose agar). To screen biosurfactant-producing yeast, the isolates were cultivated using a 96-deep well plate containing 300 μl soybean oil medium [glucose (1.5%), soybean oil (1.5%), ammonium sulfate (0.1%), potassium phosphate (0.25%), sodium phosphate (0.01%), magnesium sulfate (0.05%), calcium chloride (0.01%), manganese sulfate (0.002%) and peptone (0.1%)] at 25°C for three days. The biosurfactant production of the yeast isolates was tested using a modified drop collapse method as follows: 100 μl of the culture supernatant and water (1:1, v/v) was pipetted and placed on parafilm [20, 21]. Strain JAF-11, which was obtained from a flower (*Prunus mume* Sieb. et Zucc.) collected from an apricot village in Gwangyang in the Republic of Korea in March 2018, was selected for the further study. The strain was maintained for storage at -80°C in 15% (v/v) glycerol and was deposited with the patent depository as KACC 83047BP.

### Identification of Biosurfactant Producing Yeast

The identification of strain JAF-11 was conducted based on multigene phylogenetic analysis of the nucleotide sequences combined with the D1/D2 region of the large subunit (LSU) and the internal transcribed spacer (ITS) region ribosomal DNA (rDNA) genes. Macrogen Inc. (Korea) performed the DNA sequencing, and the gene sequences of related species were retrieved from the GenBank database. The phylogenetic tree was inferred by using the maximum likelihood method with 1,000 bootstrap replicates and sequences analysis was performed in MEGA X software (version 10.2.5, http://www.megasoftware.net).

### Time Course of the Growth and Determination of Surface Tension

A single colony of strain JAF-11 was cultivated at 25°C using a 3 ml soybean oil medium for seed culture. The seed culture was inoculated in Erlenmeyer flasks containing 500 ml soybean oil medium and cultivated at 25°C on a rotary shaker at 150 rpm. Then the optical density at 600 nm was measured daily for 8 days by a UV spectrophotometer. To confirm the production of biosurfactant, the culture medium of strain JAF-11 was prepared through a 0.22 mm filter and measured the surface tension (ST) every day. For ST measurement, the Wilhelmy plate method was used at room temperature with a force tensiometer K11 (Germany). All tests were performed in triplicates.

### Measurement of Critical Micelle Concentration (CMC) of Biosurfactant

To measure CMC of strain JAF-11, a culture (8 L) grown in soybean oil medium was mixed with Diaion HP-20 non-polar resin (Mitsubishi Chemical, Japan) and eluted in methanol. After removing methanol by rotary vacuum evaporation, the concentrated solution was partitioned with an equal volume of ethyl acetate. The ethyl acetate was concentrated, and the product was used for CMC measurement after purification using silica gel column chromatography (SK Chemical, Korea) with the solvent system, respectively CHCl_3_ : MeOH, 50:1 to 1:1 (v/v). The crude biosurfactant was dissolved in distilled water and serially diluted to concentrations of 0-250 mg/l. The CMC was determined by plotting the surface tension against the log of the biosurfactant concentration [22].

### Isolation and Purification of Biosurfactant Compound

A yeast strain of JAF-11 was cultured in the 500 ml soybean oil medium for 5 days at 25°C on a rotary shaker incubator at 150 rpm. The culture broth was subjected to HP-20 column chromatography and eluted with 30%MeOH, 70% MeOH, 100 % MeOH, and acetone. A rotary vacuum evaporator concentrated the selective MeOH fractions. The concentrate was partitioned between ethyl acetate and water. The ethyl acetate portion showing biosurfactant activity was concentrated and applied to silica gel column chromatography eluted with CHCl_3_:MeOH (50:1, 20:1, 10:1, 5:1, 2:1, and 1:1, v/v) (Fig. S1). An active fraction, CHCl_3_:MeOH (20:1), was subjected to Sephadex LH-20 (GE Healthcare, Sweden) column chromatography eluted with CHCl_3_:MeOH (1:1, v/v). The biosurfactant activity of eluates was evaluated by the drop collapse method. Fractions activity was determined by dissolving each eluate in water, loading 20 μl of the resulting solution on Parafilm, and measuring the degree of spreading. The concentrate was further separated by the medium pressure liquid chromatography (MPLC, Teledyne ISCO, USA) equipped with Redisep Rf C_18_ reversed-phase column (Teledyne Isco) using a gradient of 70%→100% aq. MeOH to yield compound 1 (13.4 mg).

### Chemical Structure Analysis of Biosurfactant

The fast atom bombardment mass spectrum (FAB-MS) and high-resolution fast atom bombardment mass spectrum (HRFAB-MS) for the molecular weight and molecular formula, respectively, were measured using a JMS-700 MStation (Jeol, Japan) mass spectrometry. In addition, the nuclear magnetic resonance (NMR) spectra were obtained on a JEOL JNM-ECZ500R, 500 MHz FT-NMR spectrometer at 500 MHz for ^1^H NMR and 125 MHz for ^13^C NMR in CD_3_OD (Andover, USA). Chemical shifts are given in ppm (δ) with tetramethylsilane as the internal standard. For NMR spectra, two-dimensional NMR such as ^1^H-^1^H correlated spectroscopy (^1^H-^1^H COSY), heteronuclear multiple quantum correlation (HMQC), and heteronuclear multiple bond correlation (HMBC) as well as one-dimensional NMR such as ^1^H NMR and ^13^C NMR were employed.

## Result and Discussion

### Isolation and Identification of Biosurfactant Producing Yeast

**Fig. 1:**
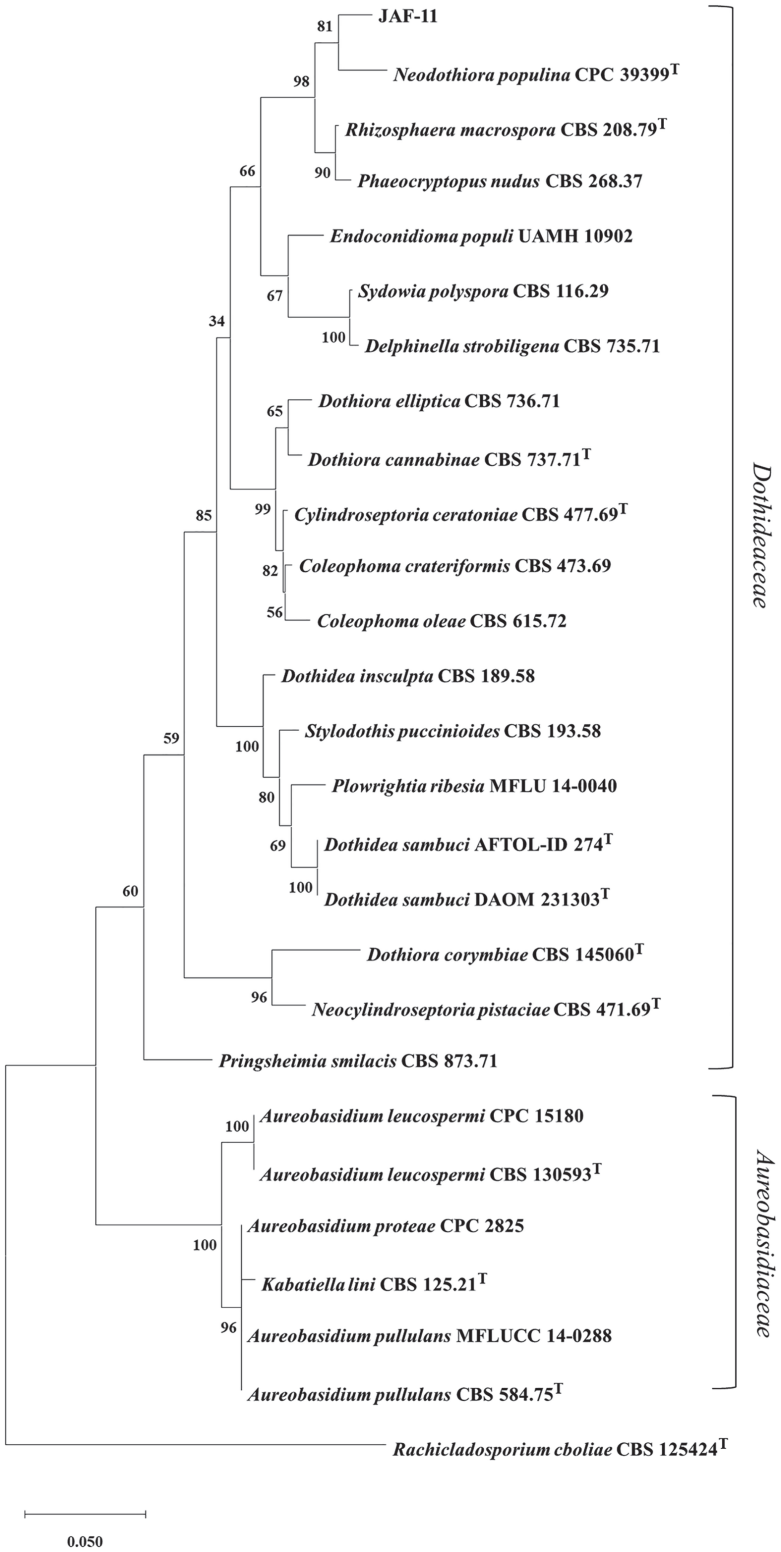
Phylogenetic tree of concatenated LSU and ITS region sequences of closely related species. *Rachicladosporium cboliae* was used as the outgroup in the phylogenetic tree. The phylogenetic tree was constructed using the maximum likelihood method and Tamura-Nei model with bootstrap values of 1,000 replicates. The scale bar indicates substitutions per nucleotide position.

Strain JAF-11 was isolated from *Prunus mume* Sieb. et Zucc. in the Republic of Korea and was selected as a potential biosurfactant producer using modified drop collapse method (Fig. S2). Phylogenetic relationships between strain JAF-11 and the closely related strains were inferred using concatenated LSU and ITS sequences [23, 24]. The reference strains LSU and ITS sequences were obtained from the GenBank database (Table 1). Phylogenetic analysis revealed that JAF-11 belonged to the family *Dothideales* clade and formed one compact cluster with *Neodothiora populina* CPC 39399^T^, *Rhizosphaera macrospora* CBS 208.79^T^ and *Phaeocryptopus nudus* CBS 268.37 (Fig. 1). The LSU region sequence of strain JAF-11 showed the highest similarity with *Rhizosphaera macrospora* CBS 208.79^T^ (97.26%; 15nt substitutions in 548 nt) and *Neodothiora populina* CPC 39399^T^ (97.75%; 13nt substitutions in 578 nt). In the ITS region sequences, strain JAF-11 had a sequence similarity of 95.15% (26 nt substitutions in 536 nt) with *Rhizosphaera macrospora* CBS 208.79^T^ and 94.27% (31 nt substitutions in 541 nt) with *Neodothiora populina* CPC 39399^T^. According to Duong Vu *et al*. (2016), the taxonomic threshold predicted to discriminate yeast species is 98.41% in the LSU region and 99.51% in the ITS region [25]. Consequently, we propose that strain JAF-11 represents a novel yeast species of a new genus based on phylogenetic analysis and the taxonomic thresholds of gene sequence identities.

### Surface-Active Properties of Biosurfactant

**Fig. 2:**
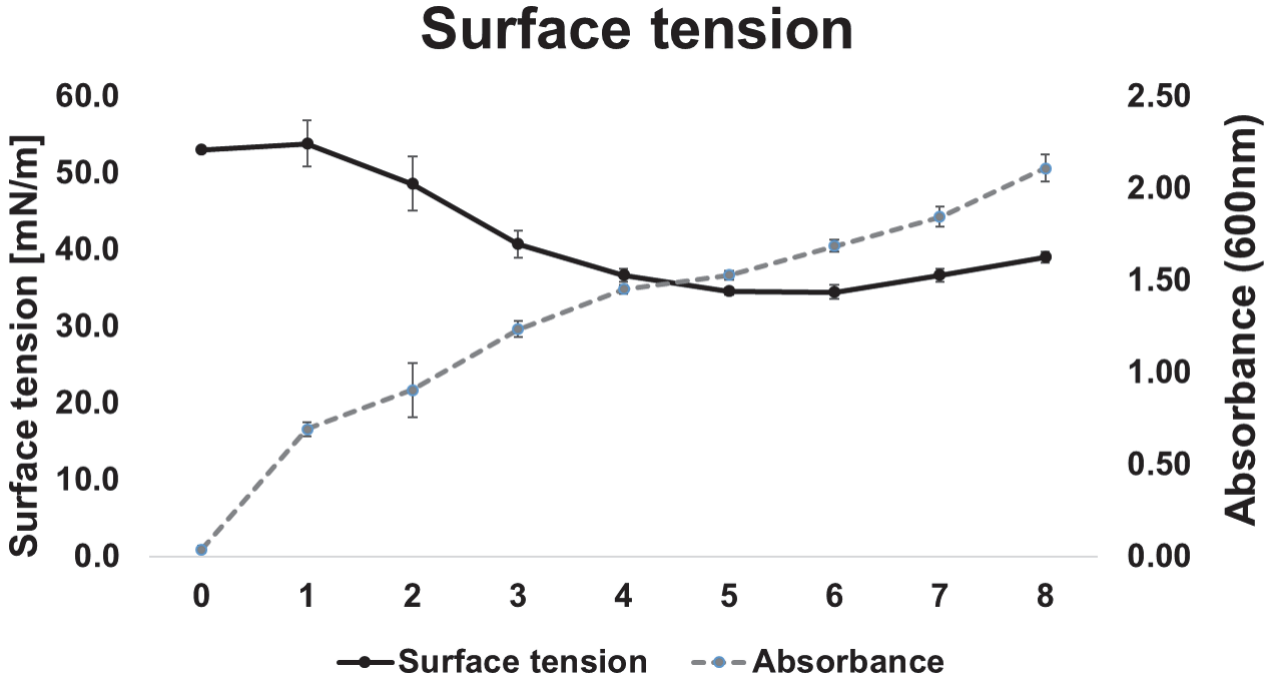
Time course of growth kinetics and surface tension in culture medium during the cultivation of strain JAF-11.

Growth of strain JAF-11 in the culture medium was detected by measuring absorbance at 660 nm and surface tensions of the aqueous supernatant were measured using force tensiometer K11. The experimental strain growth was continuously increases till 8 days, while the surface tension was decreases on 6 days (53 mN/m to 34.5 mN/m) and further increases on 7^th^ day. The surface tension recorded the lowest values of 34.5~34.6 mN/m after 5-6 days of incubation (Fig. 2). The above results indicated that the highest biosurfactant production in strain JAF-11 is reached at 5-6 days before the stationary growth phase.

**Fig. 3:**
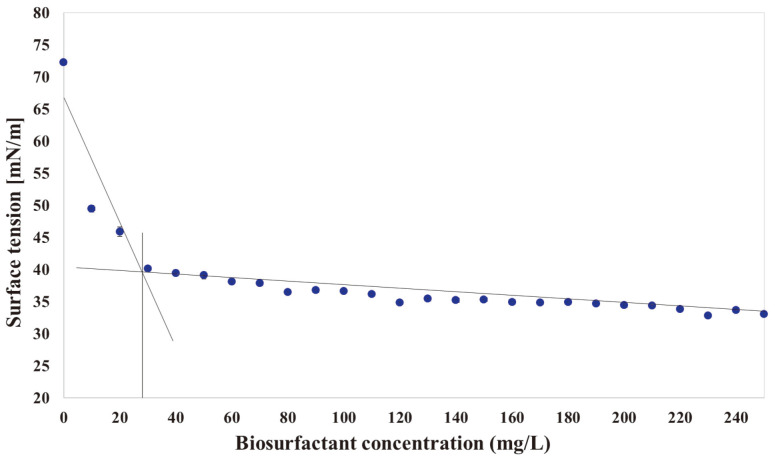
Determination of critical micelle concentration of producing crude biosurfactant from strain JAF-11.

The critical micelle concentration (CMC) is defined as the concentration of surfactant required to start the formation of the micelles. It was determined by plotting the surface tension measured according to the concentration of biosurfactant and identifying the point at which the surface tension of the biosurfactant no longer decreases dramatically. As a results of measuring the surface tension of the water with the crude biosurfactants isolated from culture medium, the values were from 72.23 mN/m to 32.80 mN/m and the minimum surface tension value was 32.80 mN/m. In particular, the value of CMC was 24 mg/l and the concentration of the biosurfactant obtained from slope of the curve abruptly changed as shown in Fig. 3.

### Chemical Structure of the Isolated Compound

**Fig. 4:**
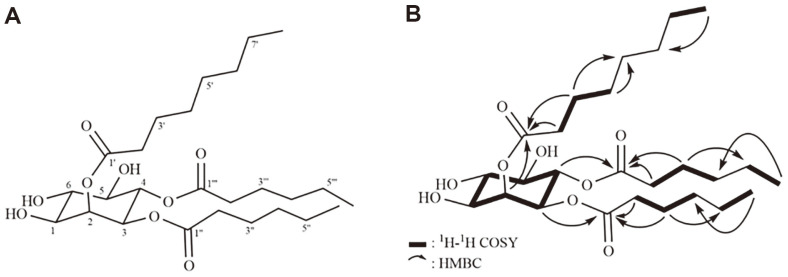
(A) Chemical structure of a new biosurfactant and (B) two-dimensional NMR correlations of a new biosurfactant.

The chemical structure of the biosurfactant isolated was determined by mass and NMR measurements. The molecular weight of 502 was determined by the FAB-MS measurement, which showed a quasi-molecular ion peak at *m/z* 503 [M+H]^+^ (Fig. S3). The molecular formula, C_26_H_46_O_9_, was determined by the HR-FAB-MS, providing a molecular ion peak at *m/z* 503.3243 [M+H]^+^ (calcd. for C26H47O9, 503.3220), indicating four degrees of unsaturation. The ^1^H NMR spectrum of 1 (Table 2) showed signals due to six oxygenated methines at δ_H_ 5.50 (t, *J* = 2.7 Hz, H-2), 5.28 (t, *J* = 10.0 Hz, H-4) , 4.93 (dd, *J* = 10.0, 2.7 Hz, H-3), 3.66 (t, *J* = 9.5 Hz, H-6), 3.65 (dd, *J* = 10.0, 2.7 Hz, H-1), and 3.45 (t, *J* = 9.0 Hz, H-5). It also showed signals attributable to 14 methylenes at δ_H_ 2.45 (m, H-2¢)/2.42 (m, H-2¢), 2.36 (m, H-2¢¢)/2.31 (m, H-2¢¢), 2.18 (m, H-2¢¢¢), 1.68 (m, H-3¢), 1.59 (m, H-3¢¢), 1.55 (m, H-3¢¢¢), and 1.35-1.25 (overlapped) and three methyls at δ_H_ 0.90 (overlapped). The ^13^C NMR spectrum (Table 2) in combination with HMQC spectrum displayed signals due to three carbonyl carbons at δ_C_ 175.0 (C-1¢), 174.6 (C-1¢¢), and 174.2 (C-1¢¢¢), six oxygenated methine carbons at δ_C_ 74.6 (C-6), 74.2 (C-5), 73.2 (C-4), 72.6 (C-2), 71.7 (C-3), and 70.9 (C-1), 14 methylene carbons at δ_C_ 23.4‒35.2, and three methyl carbons at δ_C_ 14.4 (C-6¢¢¢) and 14.2 (C-8¢ and C-6¢¢). The ^1^H-^1^H COSY correlations among six oxygenated methine protons established the presence of an inositol moiety. Inositol moiety was identified as a *myo*-inositol by the proton coupling constant. Except for an equatorial proton at δ_H_ 5.50 (H-2) with a coupling constant of 2.7 Hz, other protons occupied an axial position based on their proton coupling constants. The ^1^H-^1^H COSY spectrum also established six partial structures in three acyl chains, as shown in Fig. 4B. Chemical structure was unambiguously determined by the HMBC spectrum, which exhibited the long-range correlations. The spectrums imply the presence of two hexanoyl moieties due to long-range couplings from two methyl protons at δ_H_ 0.90 (H-6¢¢ and H-6¢¢¢) to two methylene carbons at δ_C_ 32.4 (C-4¢¢ and C-4¢¢¢), from the methylene protons at δ_H_ 1.59 to the carbonyl carbon at δ_C_ 174.6 and the methylene carbons at δ_C_ 32.4 and 23.4, and from the methylene proton at δ_H_ 1.55 to the carbonyl carbon at δ_C_ 174.2 and the methylene carbons at δ_C_ 32.4 and 23.4 (Fig. S9). The presence of an octanoyl moiety was established by the long-range correlations from the methylene protons at δ_H_ 2.45/2.42 and 1.68 to the carbonyl carbon at δ_C_ 175.0 (Fig. S9) and from the methyl protons at δ_H_ 0.90 to the methylene carbons at δ_C_ 32.9 and 23.7, and remaining two methylene carbons at δ_C_ 30.2, which showed the long-range correlations with the methylene protons at δ_H_ 1.68. Finally, the long-range correlations from the oxygenated methines at δ_H_ 5.50, 4.93, and 5.28 to the carbonyl carbons at δ_C_ 175.0, 174.2, and 174.6, respectively, revealed that C-2, C-3, and C-4 in inositol moiety were acylated with one octanoyl and two hexanoyl groups, as shown in Fig. 4B. Taken together, the structure of compound 1 was determined to be a new *myo*-inositol derivative and was named JAF-11. This compound was similar to pullusurfactan E isolated from *Aureobasidium pullulans* strain A11211-4-57 from fleabane flower, *Erigeron annus* (L.) pers. [26]. Although the chemical structure is very similar to pullusurfactan E, octanoyl moiety in compound 1 is present instead of hexanoyl moiety in pullusurfactan E (Fig. 4A). And the yeast strain JAF-11 producing the new biosurfactant is a novel yeast species that does not belong to *Aureobasidium pullulans*. Consequently, this study reports the isolation of a new biosurfactant from the novel yeast strain JAF-11 for the first time.

In conclusion, a new biosurfactant of *myo*-inositol lipid type was isolated from yeast strain JAF-11 from *Prunus mume* Sieb. *et* Zucc. The newly isolated biosurfactant has the potential to be used in industrial applications such as cosmetics, medicine, and health functional foods.

## Supplemental Materials

Supplementary data for this paper are available on-line only at http://jmb.or.kr.

## Figures and Tables

**Table 1:** GenBank accession numbers of species used in phylogenetic analyses.

Species	Strain no.	GenBank accession no.	
		LSU	ITS
Family Dothideaceae			
*Coleophoma crateriformis*	CBS 473.69	MH871117.1	MH859358.1
*Coleophoma oleae*	CBS 615.72	MH872293.1	KU728511.1
*Cylindroseptoria ceratoniae*	CBS 477.69^T^	KF251655.1	KF251151.1
*Delphinella strobiligena*	CBS 735.71	MH872074.1	MH860318.1
*Dothidea insculpta*	CBS 189.58	MH869284.1	AF027764.1
*Dothidea sambuci*	AFTOL-ID 274^T^	AY544681.1	DQ491505.1
*Dothidea sambuci*	DAOM 231303^T^	NG_027611.1	AY883094.1
*Dothiora cannabinae*	CBS 737.71^T^	MH872076.1	MH860320.1
*Dothiora corymbiae*	CBS 145060^T^	MK047482.1	MK047431.1
*Dothiora elliptica*	CBS 736.71	MH872075.1	KU728502.1
*Endoconidioma populi*	UAMH 10902	HM185488.1	HM185487.1
*Neocylindroseptoria pistaciae*	CBS 471.69^T^	MH871115.1	MH859357.1
*Neodothiora populina*	CPC 39399^T^	MW175405.1	MW175365.1
*Phaeocryptopus nudus*	CBS 268.37	GU301856.1	EU700371.1
*Plowrightia ribesia*	MFLU 14-0040	KM388552.1	KM388544.1
*Pringsheimia smilacis*	CBS 873.71	FJ150970.1	MH860390.1
*Rhizosphaera macrospora*	CBS 208.79^T^	MH872971.1	MH861202.1
*Stylodothis puccinioides*	CBS 193.58	MH869286.1	MH857753.1
*Sydowia polyspora*	CBS 116.29	MH866487.1	MH855019.1
Family Aureobasidiaceae			
*Aureobasidium leucospermi*	CPC 15180	JN712555.1	JN712489.1
*Aureobasidium leucospermi*	CBS 130593^T^	MH877257.1	KT693727.1
*Aureobasidium proteae*	CPC 2825	JN712558.1	JN712492.1
*Aureobasidium pullulans*	MFLUCC 14-0288	KM461701.1	KM388542.1
*Aureobasidium pullulans*	CBS 584.75^T^	DQ470956.1	FJ150906.1
*Kabatiella lini*	CBS 125.21^T^	FJ150946.1	FJ150897.1
Family Cladosporiaceae			
*Rachicladosporium cboliae*	CBS 125424^T^	MH875168.1	MH863703.1

**Table 2:** ^1^H and ^13^C NMR spectral data of a new biosurfactant and pullusurfactan E in CD_3_OD.

No.	Biosurfactant produced by JAF-11		Pullusurfactan E [26]	
	δ_c_	δ_H_	δ_c_	δ_H_
1	70.9	3.65 (^1^H, dd, *J* = 10.0, 2.7)^a^	69.7	3.71 (^1^H, dd, *J* = 10.0, 2.4)
2	72.6	5.50 (^1^H, t, *J* = 2.7)	70.4	5.53 (^1^H, t, br.s)
3	71.7	4.93 (^1^H, dd, *J* = 10.0, 2.7)	69.3	4.93 (^1^H, dd, *J* = 10.3, 2.7)
4	73.2	5.28 (^1^H, t, *J* = 10.0)	71.4	5.26 (^1^H, dd, *J* = 10.3, 9.6)
5	74.2	3.45 (^1^H, t, *J* = 9.0)	72.9	3.52 (^1^H, dd, *J* = 9.6, 9.6)
6	74.6	3.66 (^1^H, t, *J* = 9.5)	73.4	3.78 (^1^H, dd, *J* = 10.0, 9.6)
1'	175.0		173.5	
2'	35.2	2.42 (^1^H, m), 2.45 (^1^H, m)	34.0	2.39 (2H, t, *J* = 7.6)
3'	26.3	1.68 (2H, m)	24.6	1.62 (2H, m)
4'	30.2	1.38 (2H, m)	31.2	1.31 (2H, m)
5'	30.2	1.25-1.35 (2H, m)	22.3	1.20-1.34 (2H, m)
6'	32.9	1.25-1.35 (2H, m)	13.9	0.84-0.90(3H, overlapped)
7'	23.7	1.25-1.35 (2H, m)		
8'	14.2	0.90 (3H, overlapped)		
1''	174.6		172.8	
2''	35.1	2.31 (^1^H, m), 2.36 (^1^H, m)	33.9	2.18 (^1^H, m), 2.16 (^1^H, m)
3''	25.7	1.59 (2H, m)	24.3	1.52 (2H, m)
4''	32.4	1.32 (2H, m)	31.1	1.25 (2H, m)
5''	23.4	1.25-1.35 (2H, m)	22.2	1.20-1.34 (2H, m)
6''	14.2	0.90 (3H, overlapped)	13.8	0.84-0.90 (3H, overlapped)
1'''	174.2		173.6	
2'''	35.0	2.18 (2H, m)	34.2	2.32 (^1^H, m), 2.27 (^1^H, m)
3'''	25.4	1.55 (2H, m)	24.6	1.57 (2H, m)
4'''	32.4	1.28 (2H, m)	31.2	1.27 (2H, m)
5'''	23.4	1.25-1.35 (2H, m)	22.3	1.20-1.34 (2H, m)
6'''	14.4	0.90 (3H, overlapped)	13.9	0.84-0.90 (3H, overlapped)

^a^Proton resonance integral, multiplicity, and coupling constant (*J* = Hz) in parentheses
