# Supplementary material for: Production of a New Biosurfactant by a New Yeast Species Isolated from Prunus mume Sieb. et Zucc
Source: J Microbiol Biotechnol. 2023 May 4;33(8):1023–9. doi: 10.4014/jmb.2205.05052 (PMC10468678; doi:10.4014/jmb.2205.05052)
Supplement: Supplementary file 1 [file jmb-33-8-1023-supple.pdf]

J. Microbiol. Biotechnol.
https://doi.org/10.4014/jmb.2205.05052

Supplementry Figures

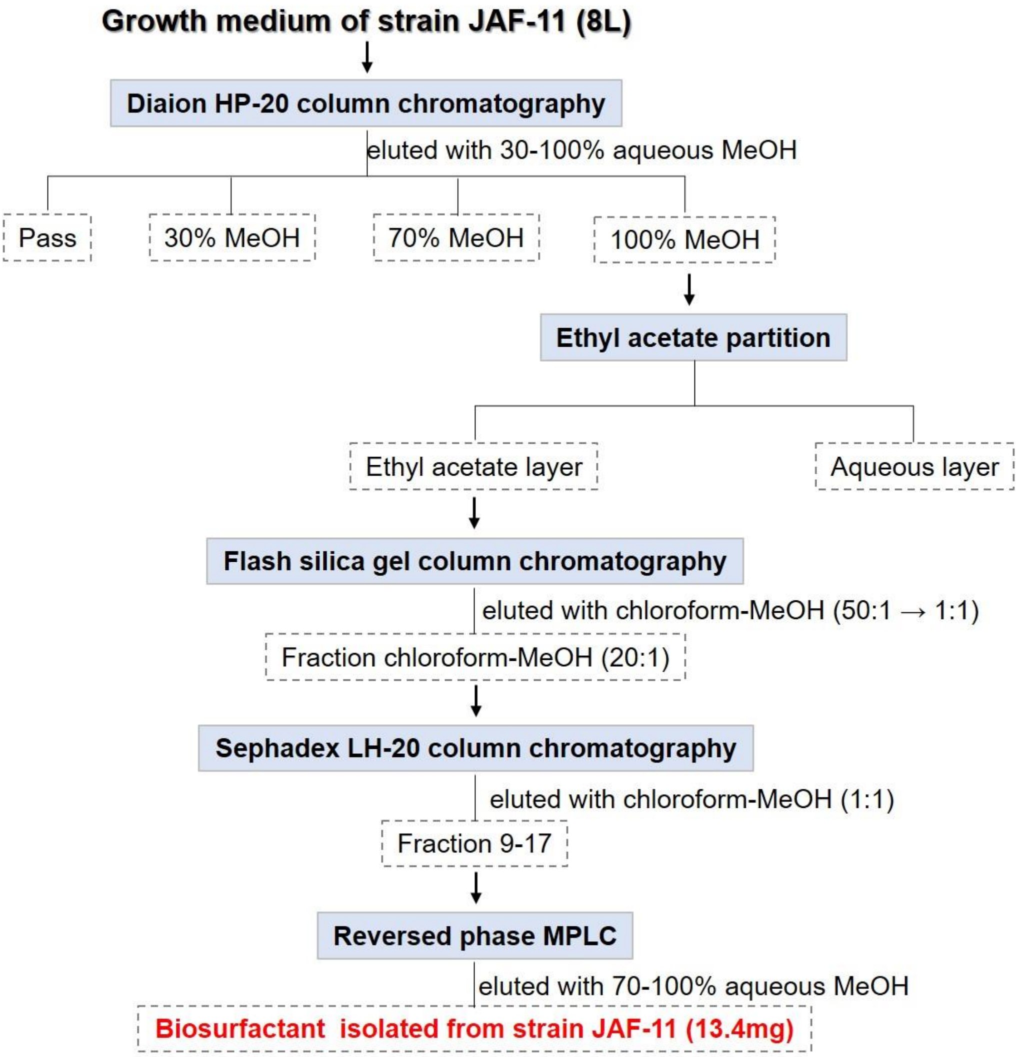

Fig. S1. Purification scheme of the biosurfactant produced by strain JAF-11.

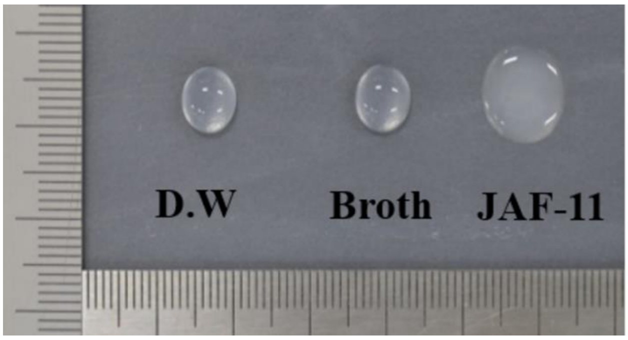

Fig. S2. Screening of biosurfactant producing yeast by drop collapse method. Distilled water and fresh culture broth were used as a control.

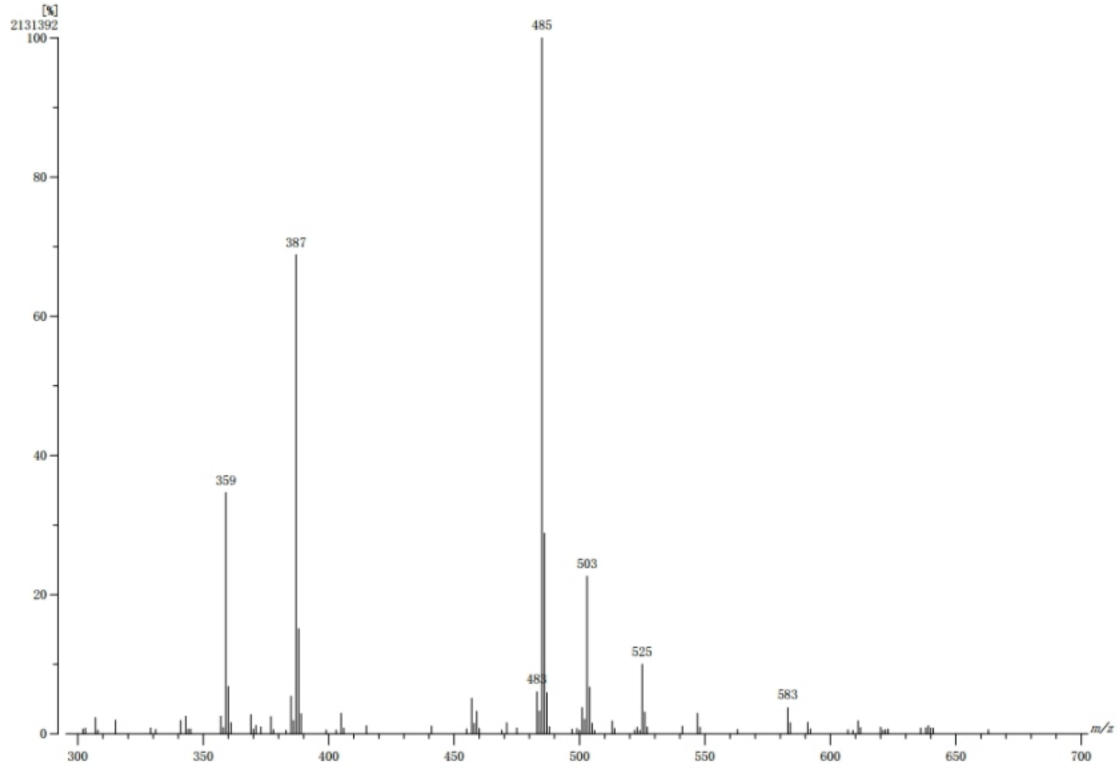

Fig. S3. Fast atom bombardment mass spectrum of purified biosurfactant in the positive ion mode.

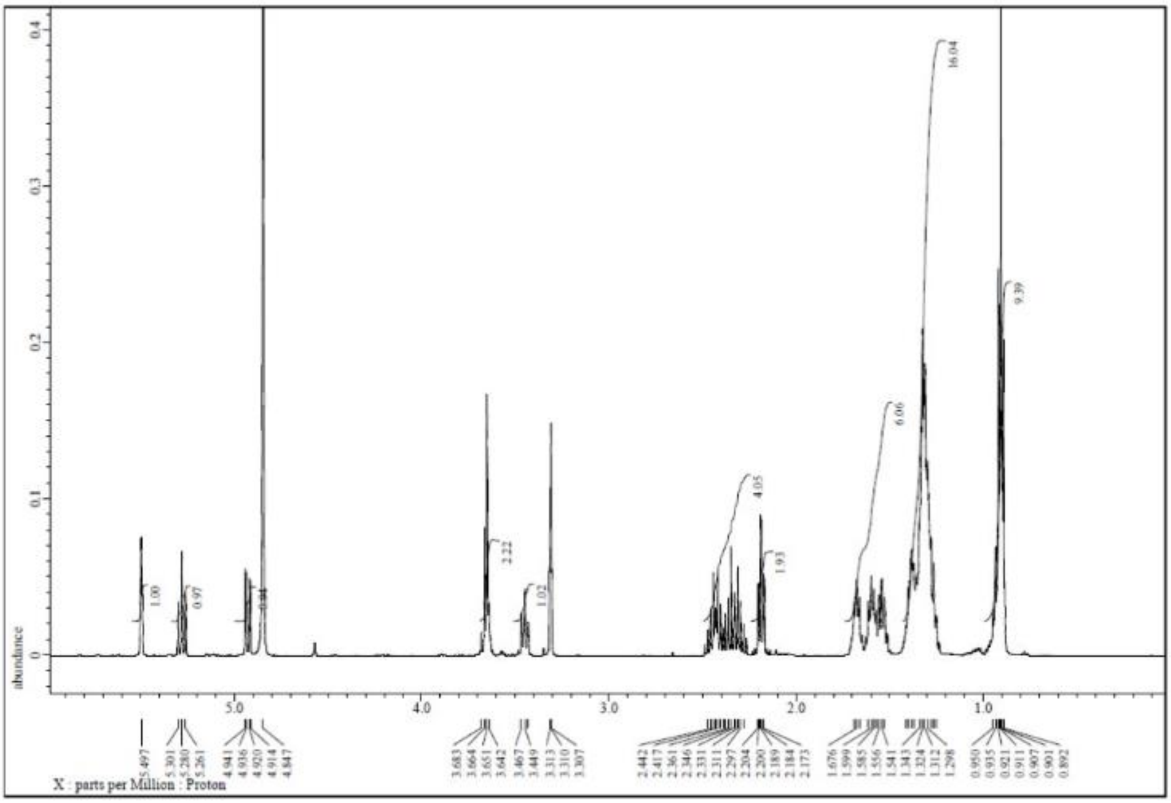

Fig. S4. ^{1}H NMR spectrum of the purified biosurfactant

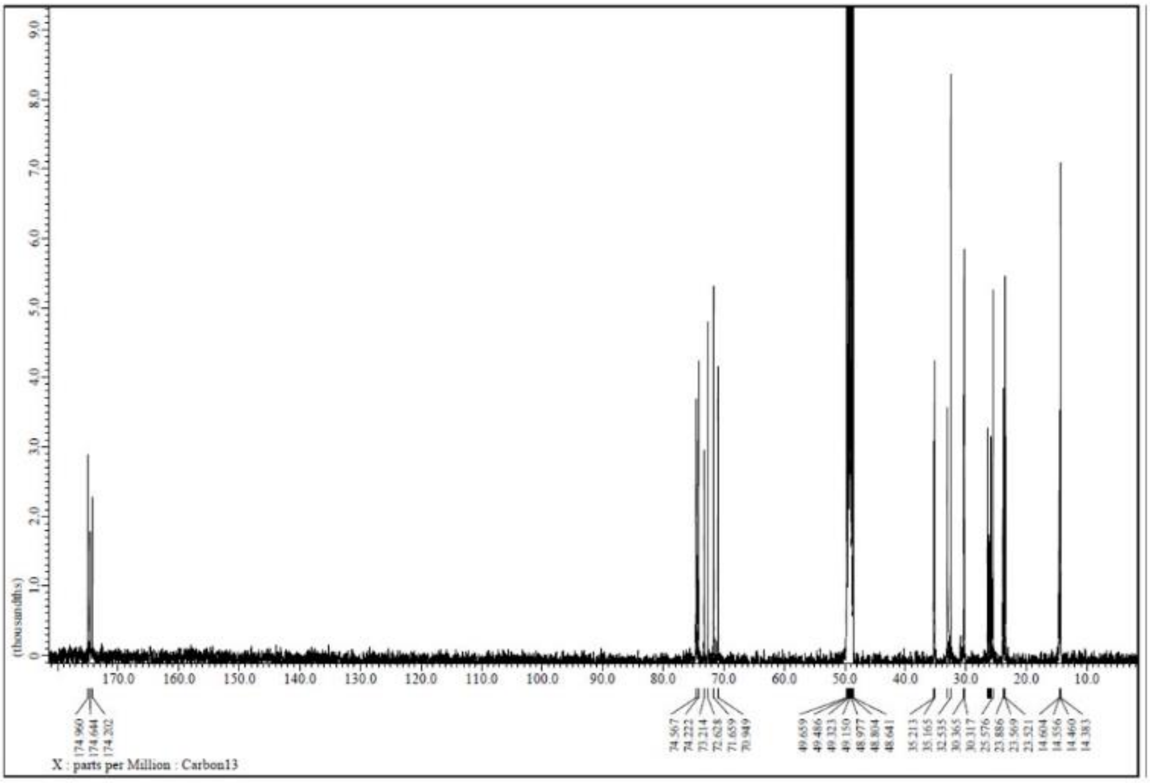

Fig. S5. ^{13}C **NMR spectrum of the purified biosurfactant.**

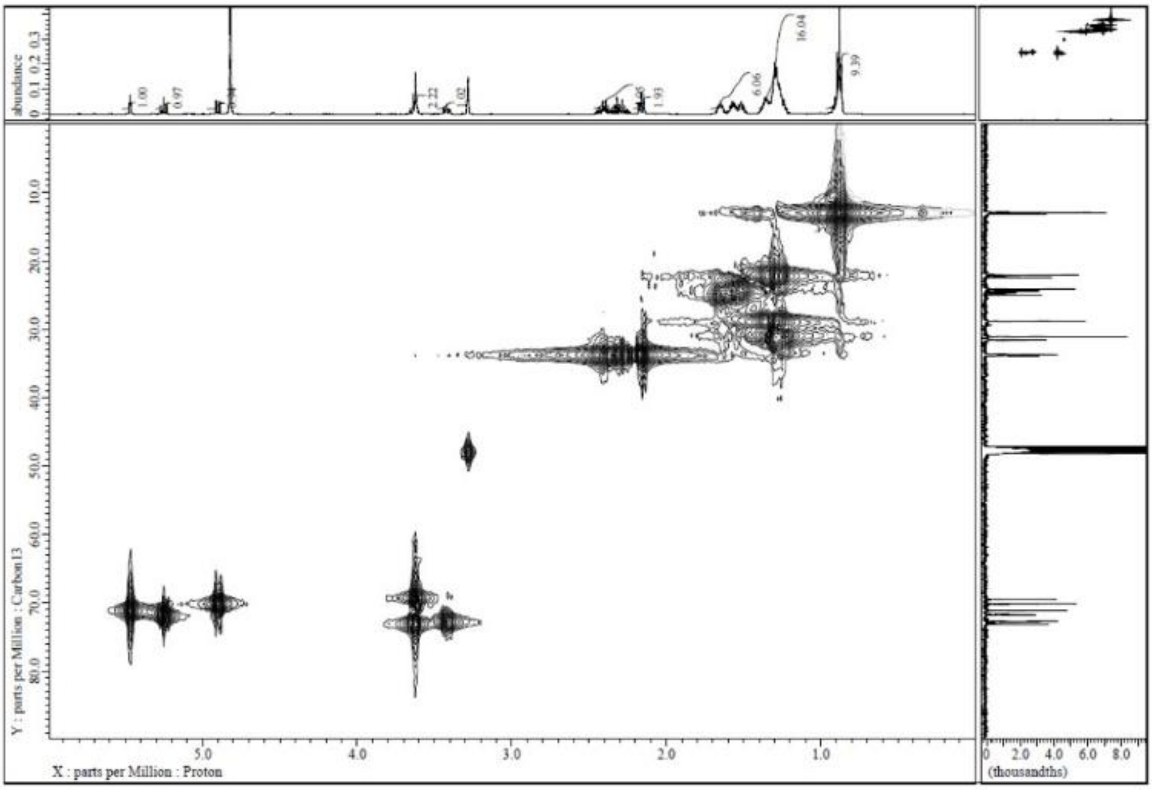

Fig. S6. HMQC spectrum of the purified biosurfactant.

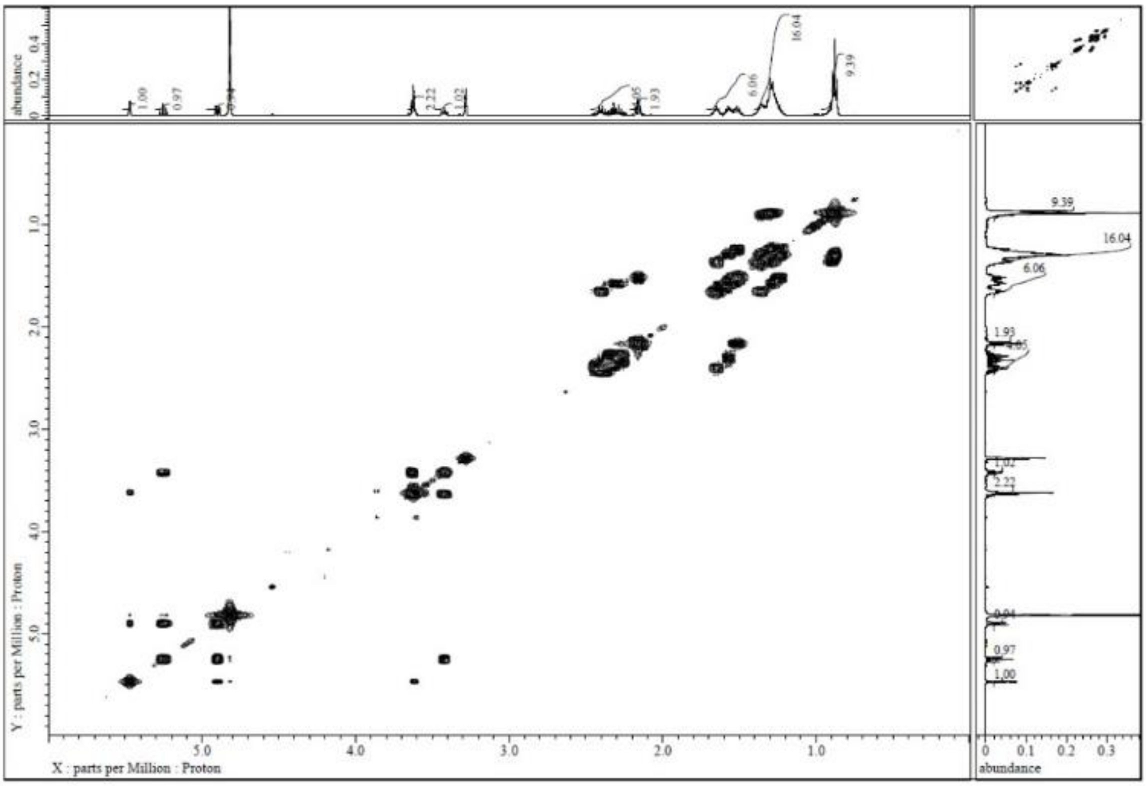

Fig. S7. ^{1}H-^{1}H COSY spectrum of the purified biosurfactant.

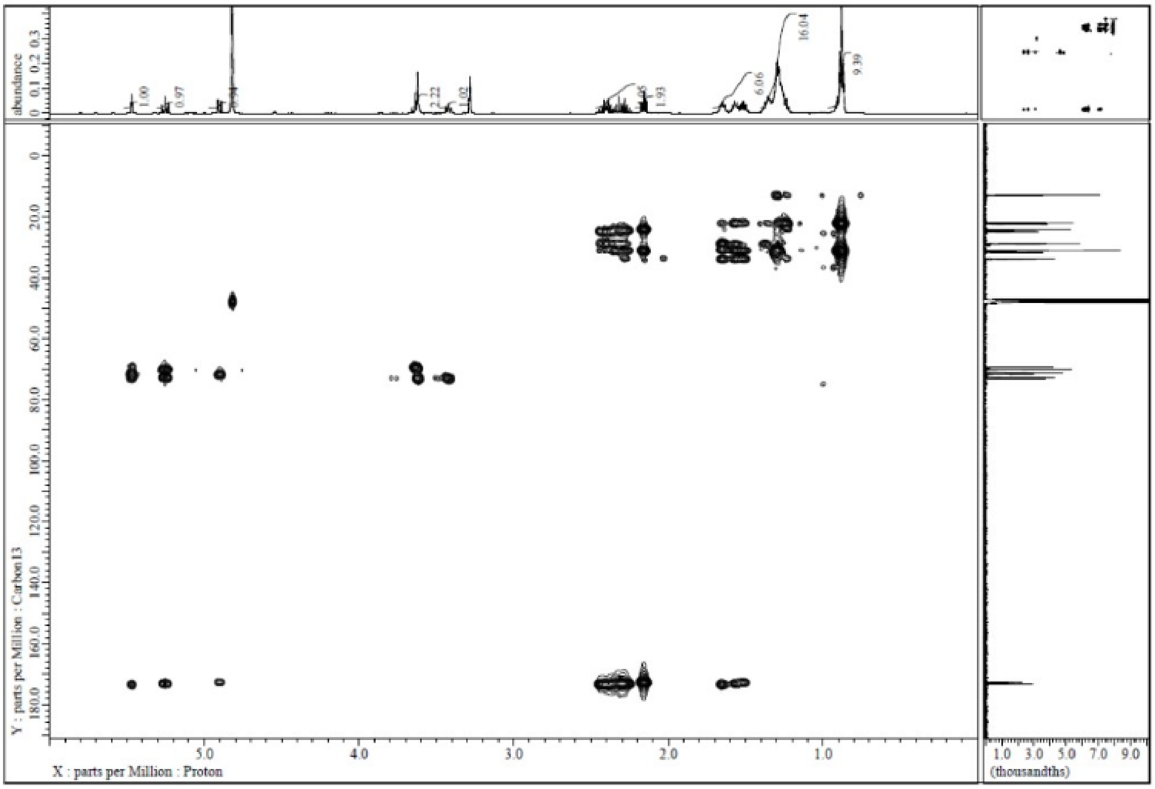

Fig. S8. HMBC spectrum of the purified biosurfactant.

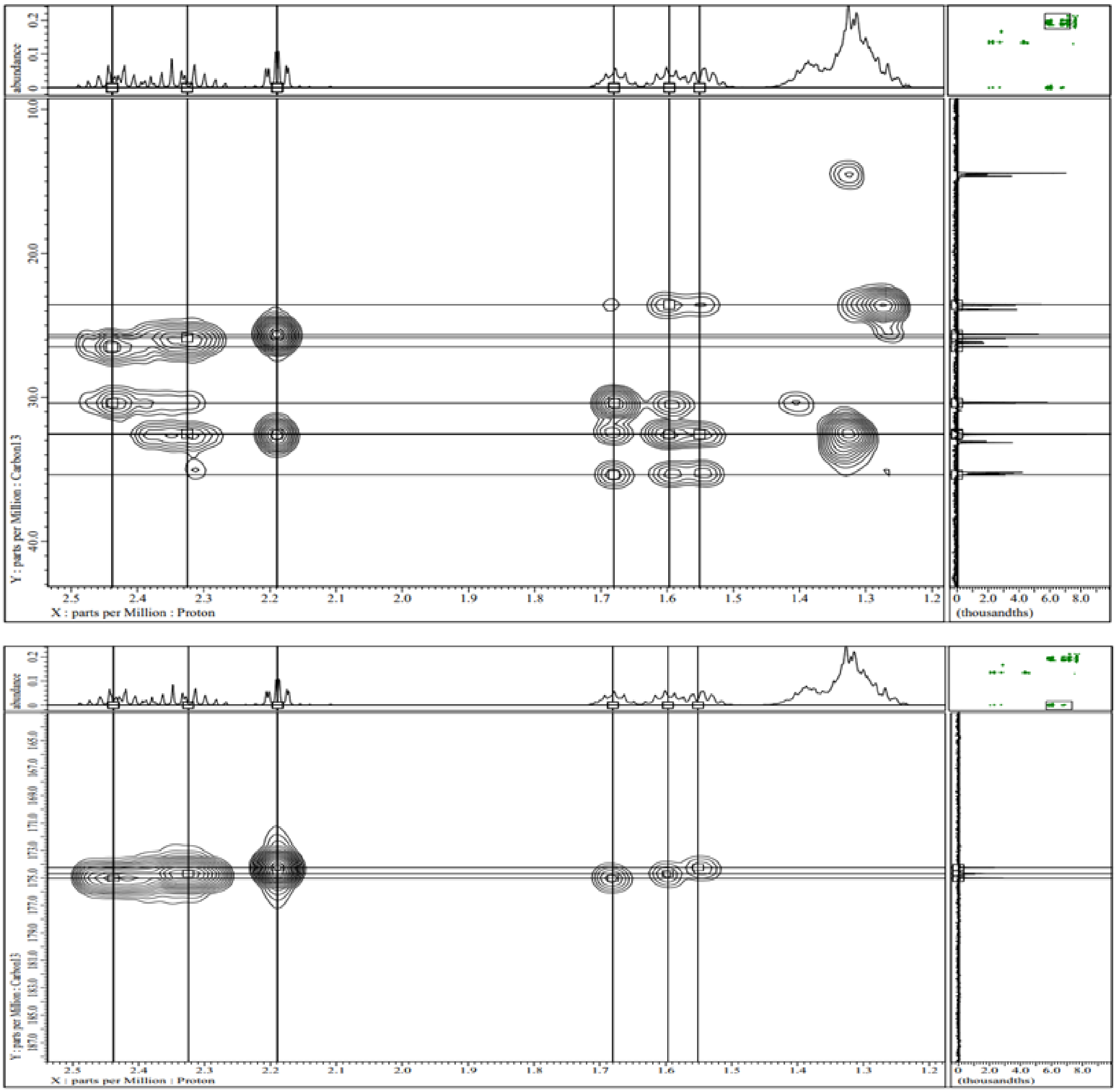

Fig. S9. Expanded HMBC spectrum of the purified biosurfactant.
